# The Role of Microorganisms in Appendiceal Pseudomyxoma Peritonei: A Review

**DOI:** 10.3390/curroncol29050289

**Published:** 2022-05-16

**Authors:** Yekaterina Khamzina, Mary Caitlin King, Carol Nieroda, D. Scott Merrell, Armando Sardi, Vadim Gushchin

**Affiliations:** 1 Department of Surgical Oncology, The Institute for Cancer Care at Mercy, Mercy Medical Center, Baltimore, MD 21202, USA; ykhamzina@nu.edu.kz (Y.K.); mking5@mdmercy.com (M.C.K.); cnieroda@mdmercy.com (C.N.); asardi@mdmercy.com (A.S.); 2 Department of Microbiology and Immunology, Uniformed Services University of the Health Sciences, Bethesda, MD 20814, USA; douglas.merrell@usuhs.edu

**Keywords:** pseudomyxoma peritonei, appendiceal cancer, cytoreductive surgery, hyperthermic intraperitoneal chemotherapy, antibacterial treatment, *Helicobacter pylori*

## Abstract

Pseudomyxoma peritonei (PMP) is a rare clinical syndrome. It originates from neoplasms of the appendix and leads to the formation of peritoneal implants and the accumulation of mucinous ascites. PMP represents a spectrum of low to high-grade disease. Despite aggressive management, many PMP patients recur, leading to debilitating symptoms and few treatment options. Therefore, scientists have continued to look for ways to improve treatment and further understand disease pathogenesis. Microorganisms were previously hypothesized to play a role in PMP progression and development. Hence, antibacterial treatment was suggested by some authors, but the data were limited. In this paper, we review the current data on the role of bacteria in PMP, discuss the significance, and suggest possible solutions to the inherent challenges in these studies. Given the limitations of the discussed studies, we remain skeptical about introducing novel antibacterial treatment into clinical practice at this time; however, the available data are valuable and indicate that more research into the molecular mechanisms of PMP is needed.

## 1. Introduction

Pseudomyxoma peritonei (PMP) is a clinical syndrome characterized by intraperitoneal mucinous spread and peritoneal implants. The condition is infrequent with an incidence of 3.2 per 1,000,000 per year [1]. Although other primaries, including the colon, ovaries, urachus, and pancreas can lead to PMP, the most common causes are perforated appendiceal low-grade mucinous neoplasms or mucinous adenocarcinomas [2,3]. PMP presentation varies from an asymptomatic incidental finding, usually after an appendectomy and/or suspected appendicitis, to abdominal discomfort, distention, increased abdominal girth, or bowel obstruction [3]. In appendiceal PMP, progressive mucin buildup eventually ruptures the appendix and spills mucus-producing cells into the peritoneal cavity [4]. Finally, the slow leak of mucus-containing cells leads to the spread of the tumor cells and the formation of implants and mucinous ascites throughout the abdomen [4,5].

Histopathologic classification of PMP has been controversial and challenging for many years [6]. Following the 2012 World Congress of the Peritoneal Surface Oncology Group International (PSOGI) in Berlin, controversial issues regarding the pathologic classification of PMP were discussed and improved definitions to resolve previous disputes were adopted [7]. Currently, PMP is classified as low-grade mucinous carcinoma peritonei (LGMCP), previously referred to as disseminated peritoneal adenomucinosis (DPAM), or high-grade carcinoma peritonei (HGMCP), previously known as peritoneal mucinous carcinomatosis (PMCA) [2]. LGMCP often has a protracted course, while HGMCP is characterized by a rapid spread [3]. Clinically this classification can be misleading, since PMP exists on a spectrum from low to high-grade and pathologic appearance may not correlate with its biological behavior [5].

With limited response to systemic chemotherapy, the treatment of choice associated with the best survival for all subtypes is cytoreductive surgery (CRS) and hyperthermic intraperitoneal chemotherapy (HIPEC) [8]. Before CRS/HIPEC became available, outcomes for PMP patients were very poor. Reports from the 1990s showed a 10-year survival of only 32% for low-grade disease [9]. However, with the use of HIPEC, a more recent systematic review from 2013 reported 3-, 5- and 10-year survival rates of 77.8%, 79.5%, and 55.9%, respectively [9]. Despite this aggressive management, high-grade disease often progresses and/or relapses leading to debilitating symptoms. Diagnosis of PMCA (HGMCP) carries an unfavorable prognosis with a median survival of 24 months and 5-year survival of 14% for the most aggressive subtypes despite appropriate treatment [10]. Since PMP carries a high risk of morbidity and mortality, the scientific community has continued to search for novel management ideas and better insights into the disease pathogenesis. Both genetic and microbial targets have been suggested. Our PMP Research Collaborative, composed of both basic scientists and clinicians, previously hypothesized that disease progression and recurrence could be due to the spillage of gut microbiota into the peritoneal cavity after appendiceal rupture; as such, antibacterial treatment in conjunction with CRS/HIPEC was considered [3,11,12,13].

Limited understanding of the disease pathogenesis, few treatment options, and high recurrence rates are the main reasons that investigators started looking into alternative ways to treat PMP. Initial research on the role of microorganisms is promising. However, we suggest that any novel findings must be interpreted with a degree of caution. In this review, we sought to provide a literature review on the role of microorganisms in PMP, antibacterial treatment, and future directions.

## 2. Bacteria and PMP

Currently, a major focus of cancer research is the role of microorganisms in carcinogenesis. More and more findings suggest that the microbiome and specific microorganisms can directly affect both tumor development and progression, as well as responses to certain therapies. The most famous example is *Helicobacter pylori*, a known risk factor for gastric adenocarcinoma and MALT lymphoma [14,15]. Other instances of bacteria facilitating or causing cancers, such as esophageal, colorectal, liver, and pancreatic, have been discussed in the literature [14,15,16]. Several contributing mechanisms, including heightened inflammation, altered gene expression, and epigenetic effect were proposed [16].

Given the growing evidence for the previously unrecognized role of microbes in cancer and the fact that the majority of PMP originates from the appendix, a known bacterial reservoir, it is natural to question whether bacteria also play a role in this disease. The first step to answering this question is to determine whether bacteria are even present in these tumors. Since a human’s peritoneal cavity is considered to be a sterile, closed environment, one would not expect to find bacteria in this location in healthy individuals. However, in instances when the appendix ruptures, as common in PMP, gastrointestinal content containing microbiota are introduced into the peritoneal cavity, which could then affect disease progression.

Semino-Mora et al. were the first to investigate the premise that gut bacteria could impact PMP pathogenesis [11]. Using in situ hybridization, Semino-Mora et al. compared the presence and load of typed and nonculturable bacteria (TNCB) between appendix samples collected from patients with PMP and those with non-inflamed, non-perforated, non-neoplastic appendixes [11]. In appendixes from PMP patients, bacteria were found inside the epithelial cells, in the network of connective tissues, and in the lumen of capillaries [11]. The distribution varied slightly between patients with DPAM (LGMCP) versus PMCA (HGMCP). Interestingly, the presence of TNCB was also observed in specimens from healthy subjects, but the distribution differed. TNCB were found in the mucus, inside epithelial and within inflammatory cells, but not on the peritoneal surface of the appendix [11]. The overall bacterial density was found to be much lower in healthy subjects compared to those with PMCA (HGMCP), but no such difference was observed between healthy subjects and those with DPAM (LGMCP) [11]. These results not only highlight the presence of bacteria in PMP tumors, but also show that the number of bacteria seems to correlate with disease severity. When testing for *H. pylori*, again using in situ hybridization, similar results were obtained. Two of five non-neoplastic, healthy subjects showed some level of *H. pylori* presence, while all DPAM (LGMCP) and PMCA (HGMCP) samples were *H. pylori* positive [11]. Similarly, *H. pylori* density was significantly higher for PMCA (HGMCP) than the other subject groups [11]. Finding *H. pylori*, a known actor in carcinogenesis, in these tumors also raises the possibility that bacteria could play a more active role in PMP. Despite these interesting results, it is important to note that the sample size was small, limited to only 5 non-neoplastic, 6 DPAM (LGMCP), and 10 PMCA (HGMCP) subjects [11]. Therefore, the true meaning behind these results remains controversial and should be further explored.

To investigate a possible relationship between these documented microorganisms and the molecular mechanisms of PMP, researchers assessed MUC2 expression. MUC2 is a gel-forming apomucin known to be associated with PMP disease [17,18]. Previous studies on this topic indicated that MUC2 protein expression could be upregulated by the lipopolysaccharide of Gram-negative organisms, such as *Pseudomonas aeruginosa* [17]. In a comparison of PMP and non-neoplastic peritoneal samples, Semino-Mora et al. found that neoplastic tissues had a much higher MUC2 expression as compared to non-neoplastic ones [11]. Moreover, there was a significant correlation between TNCB and *H. pylori* densities and MUC2 expression, suggesting that microorganisms may affect MUC2 expression [11]. The implications of these observations, however, are not yet clear. Since the study was descriptive, correlation versus causation remains unknown. Although the suggestion that MUC2 could correlate with disease severity contradicts previous findings that showed MUC2 expression to be independent of the degree of malignant transformation, it is important to note that data are limited on both sides [17]. O’Connell et al. evaluated appendix samples from 25 PMP cases and concluded that MUC2 serves as an important molecular marker of PMP, as well as a possible therapeutic target, but the expression of MUC2 does not indicate the mechanism of peritoneal spread or the degree of malignant transformation [17]. Rather, MUC2 is constitutively expressed by appendiceal goblet cells and mucin accumulation occurs because the number of MUC2-secreting cells increases and there is no place for the mucin to drain [17]. While both studies highlight the importance of MUC2 as a marker for PMP, the role of other molecular players and combinations of these markers in PMP, such as growth factors and cytokines, should not be underestimated. It was previously found that the expression of CK20 and CD44s may be related to more aggressive PMP features [19]. Given the limited and conflicting available data, further investigations regarding the cellular and molecular mechanisms of PMP are needed.

The groundbreaking discovery that enteric bacteria are indeed present in the peritoneal cavity of PMP patients opened the door for several other questions—what are these bacteria and what are they doing there? Subsequently, Gilbreath et al. conducted a study that aimed to identify the specific microorganisms in PMP samples [12]. Tumor and mucin samples from 11 PMP patients were evaluated. Numerous bacterial taxa were identified using 16S amplicon-based sequencing, direct in situ hybridization, and culturing methods (Table 1). By sequencing, the most prominent phylum identified was Proteobacteria, found in individual PMP samples with a mean abundance of 73.0%, followed by Actinobacteria (mean: 10.7%), Firmicutes (mean: 6.9%), Bacteroides (mean: 7.2%), and several others to a lesser extent [12]. Subsequently, a core microbiome was defined composed of Proteobacteria (relative abundance: 77%), Actinobacteria (relative abundance: 15%), Firmicutes (relative abundance: 5.7%), and Bacteroidetes (relative abundance: 2.3%) given that they were identified in all studied samples. Interestingly, the most abundant, Proteobacteria, has never been previously identified as a dominant phylum in healthy human gut microbiomes. It is, however, frequently found in the respiratory tract of cystic fibrosis (CF) patients [19]. Therefore, it was hypothesized that the mechanism of mucus secretion in PMP could resemble that of CF patients, which could in part explain the abundance of bacteria from the same phylum in both conditions. Indeed, an early study by Dohrman et al. in CF patients confirmed that the upregulation of MUC2 expression, the same apomucin previously discussed as an important marker in PMP, might be due to bacterial overgrowth [20]. Thus, it seems plausible that a similar bacterial-associated mechanism may contribute to PMP phenotypes. The results of the sequenced-based study were confirmed using in situ hybridization, which further highlighted the presence of certain microorganisms in PMP samples (Table 1).

The results of the culture-based analysis, which identified 11 isolates from 8 patients, are summarized in Table 1. An unclassified isolate from one patient sample, PMP191F, drew particular attention because it showed adherence and interaction with MUC2 in in vitro assays with MUC2-secreting HCT-29 cells [12]. Subsequent whole-genome sequencing revealed that PMP191F closely resembled *Niastella*, *Chitinophaga*, and *Flavitalea* genera with a definite identity. It was hypothesized that this isolate represents a novel bacteria species that might bind MUC2 and potentially influence PMP progression [21]. However, as this is based on a single isolate, it naturally requires further validation and deeper analysis. While these studies confirm the presence of microorganisms in PMP phenotypes, the results from these studies did not determine if these microorganisms are responsible for the mucin production and, hence, contributors to biological behavior, or if they simply utilize mucin as a food source to facilitate their own growth without affecting disease progression. Gilbreath et al. has also highlighted the presence of *Helicobacter* sp. in the samples from PMP patients but did not investigate this further [12]. It is important to note that the design of this study was limited to the identification of microorganisms at the phyla, genus, and order levels, but no species level identification was possible. Therefore, even though *Helicobacter* sp. was again found in PMP samples, there was not enough evidence to implicate *H. pylori* in PMP pathogenesis. However, this work did open the door for further questions about whether or not bacteria are contributors to disease phenotypes or if they simply take advantage of the mucinous environment.

## 3. Clinical Applications: Antibacterial Management of PMP

Given the evidence from previous studies documenting bacteria, including known pathogenic microbes in PMP, investigators have attempted to elucidate the impact of these enteric bacteria on disease progression. Little is known about the underlying mechanisms of PMP development, and several pathways have been proposed, but never fully elucidated. Semino-Mora et al. hypothesized that some of these enteric bacteria, including *H. pylori*, could interfere with the Wnt/β-catenin signaling pathway, a key regulator of cellular functions including proliferation, migration, and differentiation [3]. Dysregulation of the Wnt/β-catenin pathway, which is known to be influenced by *H. pylori*, has previously been linked to the progression of some cancer types, including gastric and colon cancer [14,16]. 

To investigate this, Semino-Mora et al. conducted a pilot study to assess the impact of antibiotic treatment in PMP [3]. The study involved 48 patients: 19 with DPAM (LGMCP) and 29 with PMCA (HGMCP). Overall, 14 patients were treated with combination antibiotics that are commonly used to treat *H. pylori* infections: 1 g amoxicillin, 500 mg clarithromycin, and 30 mg lansoprazole twice a day for 14 days 3 weeks prior to surgery [3]. TNCB and *H. pylori* densities, as well as β-catenin expression, in tumor samples from antibiotic-treated versus non-treated PMP patients were compared. The study concluded that antibiotic treatment resulted in a reduction in TNCB and *H. pylori* densities. This effect varied by tumor histology. Specifically, PMCA (HGMCP) patients treated with antibiotics had significantly lower bacterial densities compared to PMCA (HGMCP) patients without antibiotic therapy. Conversely, DPAM (LGMCP) patients did not show as significant a reduction. Perhaps most compelling, the study also found that β-catenin expression also varied between groups. Antibiotic-treated PMCA (HGMCP) patients showed a significant reduction in nuclear and total β-catenin levels as compared to non-antibiotic-treated PMCA (HGMCP) patients. Similar to bacterial density, the results were less pronounced in the DPAM (LGMCP) subgroup. However, the DPAM (LGMCP) subgroup also had an overall lower β-catenin expression profile as compared to PMCA (HGMCP) [3]. This suggests that bacteria and antibacterial therapy could influence disease pathogenesis. In line with this finding, Tsai et al. also highlighted the important role of the Wnt/β-cantenin pathway in the evolution of appendiceal neoplasms [22]. That study analyzed 47 appendiceal epithelial neoplasm samples for possible mutations in 11 genes. As a result, it was proposed that Wnt/β-cantenin pathway activation could be a driving force for the conversion of low-grade neoplasms into high-grade neoplasms [22]. Although there seem to be some trends and associations between bacteria, the Wnt/β-cantenin pathway, and PMP tumor progression, the causal relationship remains unconfirmed at this time and additional research into the molecular mechanisms behind the development and progression of PMP is necessary.

Interestingly, the patients included in Semino-Mora et al.’s initial antibiotics study were followed for five years and a recent article by Merrell et al. provided updated survival information on these 17 subjects enrolled in the pilot antibacterial clinical trial from 2013 [3,13]. As previously mentioned, these patients were previously treated with preoperative triple-antibiotics 3 weeks before CRS/HIPEC. Two patients initially classified as DPAM (LGMCP) were reclassified as PMCA (HGMCP). Six remaining DPAM (LGMCP) subjects were reported to be alive without disease, one alive with the disease, and one lost to follow up [13]. Survival information for the antibiotic-treated PMCA (HGMCP) subgroup was less encouraging and was influenced by lymph node (LN) status. Lymph node status was previously shown to significantly reduce the long-term survival of patients with PMCA (HGMCP), with a 5-year overall survival of 11% for LN positive versus 76% for LN negative patients (*p* < 0.001) and this trend was similar regardless of antibiotic therapy [23]. Unsurprisingly, three lymph node positive subjects succumbed to disease and died within 3.3 years; one remained alive without disease at the 125-month follow up. Among lymph node-negative PMCA (HGMCP) patients, one died from another cause, two died from the disease, and four remained alive without disease [13]. This survival information is consistent with previous studies showing that a higher disease grade correlates with a poorer prognosis [24]. Even though the initial results seemed to be encouraging, especially in lower-grade disease, the study population was too small to make a definitive conclusion about whether antibiotic treatment has any effect on PMP patient survival outcomes.

## 4. Conclusions

In this manuscript, we explored the current available data on the role of microbes in the rare, understudied disease PMP. Microbes, including known pathogenic bacteria such as *H. pylori*, have been documented in PMP tumors. Additionally, given the abundance of Proteobacteria, a known player in CF mucin production, found in PMP tumors, it is hypothesized that a similar mechanism is involved in PMP pathogenesis through the upregulation of MUC2. Similarly, it has been suggested that these microbes alter β-catenin expression/signaling through the Wnt/β-catenin pathway, which has been implicated in both PMP microbiome and tumor genomic analyses. However, conflicting and insufficient data make it difficult at this time to discern whether the bacteria play an active role in disease behavior or are simply taking advantage of a mucinous environment. The idea that bacteria could be potential targets for supplemental treatment is interesting, especially in a disease with few treatment options; however, the initial results from the pilot antibiotic treatment study combined with the fact that the role of bacteria in PMP initiation and progression is in the early stages of investigation limits the current clinical application. We hope that this review serves to draw attention to the need for additional research into the molecular mechanisms of PMP. Work in this area could help to identify additional treatment options for targeted therapies and improve outcomes for patients, especially after CRS/HIPEC fails.

There are several challenges faced by investigators trying to elucidate the molecular mechanisms of this rare tumor. One of the major hurdles is small sample size. Taking into account that the disease is relatively rare, partnership between HIPEC centers could help to address this issue. Another important factor is the evolving classification of these tumors. Several studies documented microbiologic differences in DPAM (LGMCP) and PMCA (HGMCP); however, as these tumors exist on a clinical spectrum, it can be difficult to correctly group and study these groups individually. While these challenges are faced by both clinical and basic science researchers, close collaboration, and pathologic review by PMP specialists is essential to limit confounding variables, especially when working with small populations. In addition, we believe that more careful taxonomic identification of bacteria is needed to be able to target disease-specific species if they exist. In this case, accounting for possible sample contamination during surgery, transfer and storage, and proper sampling are crucial. Careful protocol planning in partnership with bench scientists might be helpful. Currently, the only tested antibacterial regimen for PMP mainly targeted *H. pylori*. However, the data supporting the presence of this organism are still limited, raising a question about the relevance of this treatment. Another important aspect that needs to be considered before attempting to adjust PMP treatment protocols is careful examination of the existing technique. The chemotherapy drug most commonly used during HIPEC treatment, mitomycin-C, already possesses antibacterial properties. Therefore, combined with heat, it is possible that CRS/HIPEC with mitomycin-C eliminates bacteria in the peritoneal cavity without the need for additional antibacterial treatment at the time of initial therapy. Overall, we believe that novel treatment ideas for PMP should be investigated; however, they cannot be adopted into clinical practice until more solid evidence-based results are obtained.

In conclusion, PMP is a complex syndrome with a not fully understood molecular nature. There is still an ongoing discussion regarding the mechanisms of development and progression, the role of cellular and molecular pathways, as well as additional treatment strategies for PMP. In the past, understanding the genetic and molecular principles of certain cancer types helped to develop targeted therapies that improved survival. In the case of PMP, there are still many grey areas that need to be addressed before such treatments can be introduced. Considering the available information from different studies, summarized in Table 2, we believe that there is not enough evidence to recommend antibacterial treatment for PMP patients at this time; the role of bacteria in PMP disease development and progression is yet to be fully determined. Given the promising findings so far, more basic science research should be conducted to prove a stronger association between PMP and bacteria before proposing antibacterial treatment for these patients. However, there is no doubt that these initial studies have helped to lay the groundwork for further studies that are needed to define the role of bacteria and the molecular mechanisms of this deadly disease.

## Figures and Tables

**Table 1:** Microorganisms identified and/or isolated from PMP tumors and mucin in Gilbreath et al. [12].

	Culturing/Isolation Method		
**Microorganism Classification Level**	16S Sequencing	In Situ Hybridization (ISH)	Culture
Phylum	**Proteobacteria** **Actinobacteria** **Firmicutes** **Bacteroidetes**	* Proteobacteria **Actinobacteria** **Firmicutes** **Bacteroidetes**	-
Order	-	Verrucomicrobiales Rhizobiales	-
Genus	** *Methylobacterium* (106,626 seq) *Variovorax* (10,4621 seq) *Escherichia_Shigella* (88,823 seq) ***Propionibacterium*** **(81,731 seq)** ***Pseudomonas*** **(64,037 seq)** *Tessaracoccus* (41,711 seq) *Acinetobacter* (35,628 seq) *Helicobacter* (33,441 seq) ***Streptococcus* (17,987 seq)** *Acidovorax* (15,911 seq) *Moraxella* (8777 seq)	***Pseudomonas*** ***Propionibacterium*** ***Streptococcus*** sp.	*** Propionibacteriaceae, ***Propionibacterium*** (PMP196, PMP213, PMP219, PMP229, PMP267-3) Corynebacteriaceae, *Corynebacterium* (PMP238, PMP267B) Chitinophagaceae (unclassified), *Niastella* (PMP191F) Bradyrhizobiaceae, *Bosea* (PMP191M) Dermacoccaceae, *Dermacoccus* (PMP191C) Pseudonocardiaceae, *Amycolatopsis* (PMP215)

* Including Betaproteobacteria and Gammaproteobacteria; ** Top 11 most frequent genera listed of 34 identified; *** Listed as Family, Genus (Patient/Sample ID). Bold terms denote microorganisms matched across methods. Seq sequences; sp. species.

**Table 2:** Summary of studies investigating bacteria and PMP.

Study	Main Findings	Limitations
Semino-Mora et al. (2008) [11]	Enteric bacteria identified in PMP appendix samples Bacterial presence and MUC2 expression were higher in PMCA (HGMCP) than in DPAM (LGMCP) or controls MUC2 expression correlates with bacterial densities	- Small sample size (*n* = 10 PMCA (HGMCP), *n* = 6 DPAM (LGMCP), *n* = 5 controls) - Cause and effect could not be determined
Gilbreath et al. (2013) [12]	Dominant phyla were determined to include Proteobacteria, Actinobacteria, Firmicutes, and Bacteroides Some identified bacteria could interfere with MUC2 Pilot study using antibacterial treatment initiated	- Small sample size (*n* = 11) - No species-level identification - Cause and effect could not be determined - Survival results are controversial due to small sample size
Semino-Mora et al. (2013) [3]	Higher *H. pylori* densities in PMCA (HGMCP) vs. DPAM (LGMCP) Antibiotic-treated PMCA patients had significantly lower bacterial densities and decreased nuclear and total β-catenin levels	- Role of *H. pylori* and Wnt/β-catenin pathway in PMP was not investigated - Cause and effect could be determined - Antibiotic regimen may not have been optimized as some bacteria remained alive post-therapy
Merrell et al. (2019) [13]	Reported some survival differences between antibiotic treated and non-antibiotic treated PMP patients	- Small sample size (*n* = 17) - Evolving histopathologic classification made elucidating benefit in subgroups challenging - True survival benefit impossible to quantify

## Data Availability

Not applicable.
